# Computational profiling and prognostic modeling based on lysosome-related genes in colorectal cancer

**DOI:** 10.3389/fgene.2023.1203035

**Published:** 2023-11-23

**Authors:** Linjie Zhang, Jingbang Yang, Yizhang Deng, Wuguo Deng, Liren Li

**Affiliations:** ^1^ Department of Colorectal Surgery, Sun Yat-sen University Cancer Center, Guangzhou, China; ^2^ State Key Laboratory of Oncology in South China, Collaborative Innovation Center for Cancer Medicine, Sun Yat-sen University Cancer Center, Guangzhou, China

**Keywords:** colorectal cancer, lysosome related genes, immunotherapy, TME, bioinformatic

## Abstract

**Background:** Despite significant advances over the past decade, patients diagnosed with advanced colorectal cancer (CRC) continue to face unfavorable prognoses. Recent studies have underscored the pivotal role of lysosomes in tumor development and progression. This led us to postulate and develop a novel lysosomal-centric model for predicting CRC risk and therapeutic response.

**Methods:** CRC tissue samples were sourced from the TCGA database, while lysosome-associated genes were collated from the GSEA database. Differentially expressed lysosome-related genes (DE-LRGs) were discerned by contrasting tumor samples with normal tissue. Based on the expression profile of DE-LRGs, patients were stratified into two distinct clusters. Survival disparities between the clusters were delineated using Kaplan-Meier estimators. For tumor microenvironment assessment, we employed ESTIMATE and ssGSEA. Functional pathway enrichment was ascertained using both GSVA and GSEA. Subsequent uni- and multi-variate Cox regression analyses pinpointed risk-associated DE-LRGs. Leveraging these genes, we constructed a novel risk prediction model and derived risk scores. The model’s prognostic capability was externally validated using dataset GSE39084. The mutational landscape across risk categories was evaluated using the Maftools algorithm. The potential efficacy of targeted and immunotherapeutic interventions for each patient cohort was gauged using pRRophetic, CYT, and IMvigor210.

**Results:** We identified 46 DE-LRGs. Tumor Immune MicroEnvironment (TIME) assessment revealed that cluster 2 patients exhibited elevated ESTIMATE, Immunocore, and stromal scores, yet diminished tumor purity relative to cluster 1. Notable differences in immune cell infiltration patterns were observed between clusters, and distinct pathway enrichments were evident. Cluster 2 manifested a pronounced expression of immune checkpoint-related genes. Four DE-LRGs (ATP6V0A4, GLA, IDUA, and SLC11A1) were deemed critical for risk association, leading to the formulation of our novel risk model. The model exhibited commendable predictive accuracy, which was corroborated in an external validation cohort. A palpable survival advantage was observed in high-TMB, low-risk subgroups. Moreover, the low-risk cohort displayed heightened sensitivity to both targeted and immunotherapeutic agents.

**Conclusion:** Our findings underscore the potential of lysosome-associated genes as robust prognostic and therapeutic response markers in CRC patients.

## Introduction

Colorectal cancer (CRC) is a leading gastrointestinal malignancy, holding the third position in global cancer-related morbidity and the second in cancer-associated mortality (Torre et al., 2015). Adenocarcinoma dominates the histopathological landscape, constituting about 95% of CRC diagnoses, with squamous cell and mucinous carcinoma filling the remaining 5%. Early-stage diagnosis offers a promising 5-year overall survival rate surpassing 90% (Dekker et al., 2019). However, the paucity of robust diagnostic tools often results in diagnostic delays. Advances in chemotherapy and targeted therapies have enhanced treatment paradigms, yet patients with metastatic CRC (mCRC) confront a daunting prognosis, attributed to heightened recurrence and drug resistance. Specifically, advanced CRC presents with over a 40% recurrence rate, and the 5-year survival rate for mCRC remains below 20% (Andre et al., 2020; Biller and Schrag, 2021).

Recent innovations spotlighting immunotherapy have shown remarkable efficacy, particularly in CRC patients exhibiting dMMR/MSI-H, with disease-free survival (DFS) rates between 70% and 90% (Ganesh et al., 2019; Diaz et al., 2022). However, dMMR/MSI-H characterizes only 15%–30% of the general CRC population and a mere 2%–4% in mCRC, relegating a significant majority with MMR/MSS, a “cold” tumor phenotype resistant to immunotherapy (Ganesh et al., 2019). Given this landscape, there is a pressing demand for innovative models for predicting survival and therapeutic outcomes in advanced or metastatic CRC.

Lysosomes have been recognized as pivotal cellular organelles, steering myriad processes from protein secretion and endocytic receptor recycling to energy metabolism and intricate cell signaling pathways (Perera and Zoncu, 2016; Piao and Amaravadi, 2016; Ballabio and Bonifacino, 2020). The cathepsin protease family, featuring over 60 hydrolases, garners significant research attention. Particularly, cathepsins B, S, and E have associations with cancer dynamics, with their cellular localization determining their tumorigenic roles (Piao and Amaravadi, 2016). Moreover, lysosomal membrane proteins are emerging as key players in modulating cell-cell adhesion and migration, especially in the context of metastatic CRC (Piao and Amaravadi, 2016).

Recent investigations underscore the pivotal roles of lysosomes in apoptotic, autophagic, and degradative pathways, with a direct nexus to CRC’s onset and progression (Rizzollo et al., 2021; Wang et al., 2021). Autophagy, in tandem with lysosomes, has been linked to CRC tumor progression, angiogenesis, and chemoresistance (Zhao et al., 2020; Chen et al., 2022; Yang et al., 2022), positing it as a potential therapeutic target (Fu et al., 2020; Yang et al., 2022). Crucially, lysosomes have been implicated in modulating immunotherapy efficacy through their involvement in PD-L1 degradation (Gou et al., 2020).

**FIGURE 1:**
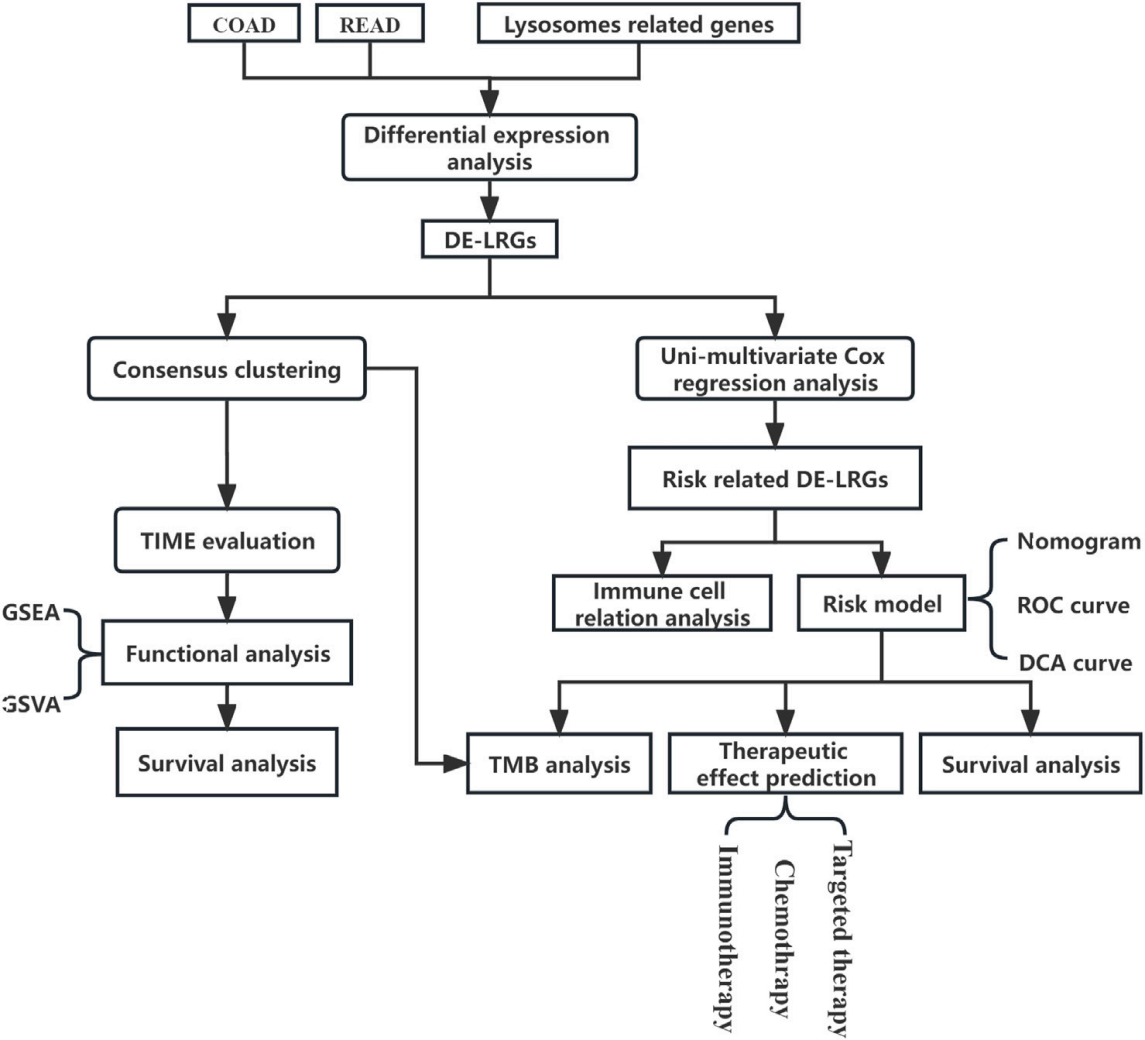
Flowchart of research.

In this study, we probed genomic shifts in CRC patients, harnessing data from the Cancer Genome Atlas (TCGA) and Gene Expression Omnibus (GEO). Our comprehensive analysis of lysosome-associated genes revealed molecular subtypes in CRC with potential as predictive biomarkers for clinical-pathological attributes and patient prognosis. We further discerned associations between these molecular subtypes, stromal activity in the tumor microenvironment, tumor-infiltrating immune cells (TIICs), and immune checkpoints. Notably, we introduced a scoring system anchored on lysosome-associated genes, designed to enhance the prediction of patient outcomes and the efficacy of CRC immunotherapy. The process of this study was shown in Figure 1.

## Materials and methods

### Patient cohort and data acquisition

In this study, we used The Cancer Genome Atlas (TCGA) database as the training cohort and GSE39084 as the validation cohort. Their clinical details were shown in Supplementary Table S2.

RNA-sequencing data from 698 CRC samples (including COAD and READ) were sourced from TCGA database. This dataset encompassed 647 tumoral and 51 adjacent non-tumoral samples. Comprehensive clinicopathological metadata accompanied these samples (https://portal.gdc.cancer.gov/).

### Identification of lysosome-associated genes

A curated list of 163 lysosome-associated genes was retrieved from the Gene Set Enrichment Analysis (GSEA) database (https://www.gsea-msigdb.org) for subsequent analyses (Supplementary Table S1).

### Analysis of differential-expressed lysosomes-related Genes (DE-LRGs)

DE-LRGs were discerned by contrasting the transcriptomic profiles of 51 normal tissues against 647 COAD and READ samples from TCGA. Stringent filtering criteria of |log_2 Fold Change| > 1 and FDR <0.05 were applied. Protein-protein interactions among these DE-LRGs were mapped using the STRING database.

### Molecular subtyping through consensus clustering

The “ConsensusClusterPlus” R package (Wilkerson and Hayes, 2010) facilitated molecular subtyping based on DE-LRG expression profiles. Parameters encompassed 80% item resampling, a maximum K of 5, 50 iterations, and 1-Pearson correlation distances. Clusters ranging from K = 2 to K = 5 were assessed, with the optimal cluster number determined through cumulative distribution function (CDF) and consensus heatmaps.

### Tumor Immune Microenvironment (TIME) and Immune Infiltration Analysis.

The ESTIMATE algorithm was employed to compute stromal and immune scores, as well as tumor purity, leveraging gene expression data (Yoshihara et al., 2013). Additionally, single-sample gene set enrichment analysis (ssGSEA) assessed immune cell enrichment in both identified clusters.

### Functional pathway enrichment

Gene set variation analysis (GSVA) discerned differential signaling pathway activations between the clusters. The “GSVA” R package facilitated this, while a subsequent GSEA provided deeper insights into inter-cluster differences.

### Gene expression profiling

Distinctive DE-LRG expressions across clusters were assessed, alongside the expression of immune checkpoint-related and HLA family genes. Results were visualized using boxplots.

### Survival outcomes and prognostic model development

Prognostically relevant DE-LRGs were delineated using univariate and multivariate Cox regression. Kaplan-Meier analysis compared survival outcomes between clusters. A risk model, evaluated through time-dependent ROC analysis using the “survivalROC” package, was then established, with its robustness verified using the GSE39084 cohort. Clinical data of the corhort was shown in Supplementary Table S2.

### Risk model formulation and validation

Risk models, based on previously identified genes via Cox regression, were formulated. Risk scores were ascertained for each patient, categorizing them into risk groups based on median values. Cox regression analyses ascertained risk factors, culminating in a nomogram. ROC and calibration curves gauged model prediction accuracy, complemented by Decision Curve Analysis (DCA).

### Mutational landscape and TMB assessment

Using “maftools”, mutational landscapes were charted, and oncoplots for risk categories were produced. Tumor mutation burden (TMB) was determined using the same package (Mayakonda et al., 2018), and differential mutational landscapes between risk categories underwent Fisher’s exact test scrutiny, with subsequent survival analysis via K-M plotter.

### Chemotherapeutic and immunotherapeutic response prediction

To forecast IC50 values of prevalent chemotherapeutics, the “pRRophetic” package was utilized (Geeleher et al., 2014). The Wilcoxon test discerned group differences. The Tumor Immune Dysfunction and Exclusion (TIDE) algorithm estimated the TIDE score and forecasted immune checkpoint blockade responses (Jiang et al., 2018).

### Cytolytic activity score (CYT) calculation

From the TCGA database, FPKM values were transmuted to TPM values. Cytolytic activity, indicative of CD8^+^ T cell activation, was calculated based on GZMA and PRF1 transcript levels (Rooney et al., 2015).

### Immunotherapy response analysis

An anti-PD-L1 immunotherapy cohort of CRC patients was accessed from the IMvigor 210 Core Biologies R package (Mariathasan et al., 2018). Risk scores allocated patients into risk categories, with subsequent analyses assessing therapeutic efficacy. Information on the response to immunotherapy was obtained for each patient and classified into one of four categories: stable disease (SD), partial response (PR), progressive disease (PD), or complete response (CR). Risk scores allocated patients into risk categories, with subsequent analyses assessing therapeutic efficacy.

### Cell culture

Colon cancer cell lines, HCT-116, SW-480, and HCT-8, alongside the standard colon epithelial line FHC, were procured from the American Type Culture Collection (ATCC; http://www.atcc.org/). These cells were propagated in RPMI-1640 medium enriched with 10% fetal bovine serum (FBS), 100 mg/mL penicillin, and 100 mg/mL streptomycin. Upon reaching approximately 80% confluence, the cells were subcultured post-dissociation with 0.25% trypsin-EDTA. Culture maintenance was performed at 37°C in a 5% CO2 atmosphere. For specific experimental conditions, cells were subjected to a hypoxic environment, consisting of 94% N2, 5% CO2, and 1% O2.

### Quantitative real-time PCR assay

The qRT-PCR assay was employed to elucidate the expression variances of risk-associated DE-LRGs between colon cancer cells and their normal counterparts. RNA from the designated cells was harvested using the RN001 RNA Quick Purification kit (ESscience). This RNA served as a template for the synthesis of first-strand cDNA via the same kit. The qRT-PCR reactions were orchestrated on the Bio-Rad SPX platform (either 96-well or 384-well format), utilizing a 2X SYBR Green mix from Life Technologies (Carlsbad, CA, United States). Expression data were normalized against GAPDH levels for analytical consistency.

## Results

### Differential expression and PPI network of LRGs

**FIGURE 2:**
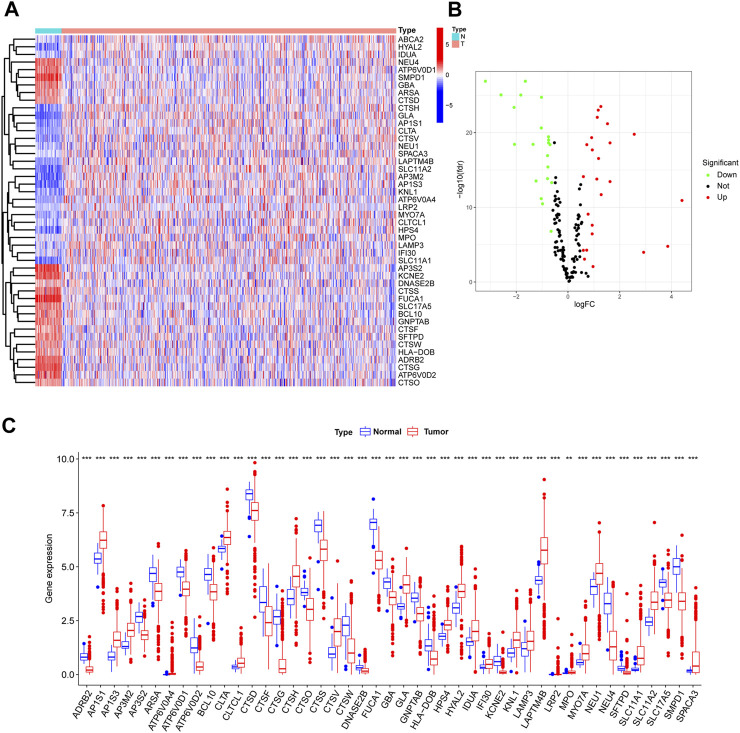
Differential expression analysis of lysosme related genes in CRC based on TCGA. **(A,B)** Heatmap and Volcano plot shown the differential expression of LRG of normal and tumor samples. **(C)** Barplot shown expression level of DE-LRGs.

Visualization of LRGs differential expression between normal and tumor samples from TCGA was achieved through a heatmap (Figure 2A). This revealed 46 distinct DE-LRGs, with 24 being upregulated and 22 being downregulated in the tumor cohort (Figure 2B). The significant differential expression of these DE-LRGs is represented in Figure 2C. A subsequent analysis highlighted the correlation and protein-protein interaction (PPI) dynamics among these DE-LRGs (Supplementary Figure S1).

### Molecular subtyping based on DE-LRGs reveals two distinct clusters

**FIGURE 3:**
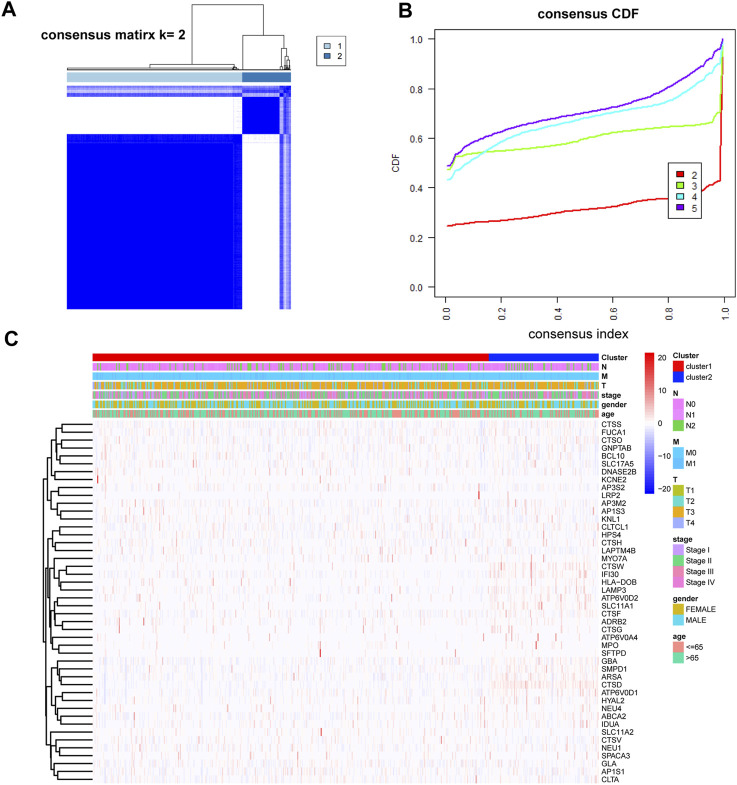
Identification of molecular subtypes based on DE-LRGs. **(A,B)** K = 2 was identified as the optimal value for consensus clustering. **(C)** Heatmap shown DE-LRGs expression in different clusters.

Using the consensus clustering paradigm predicated on the 46 DE-LRGs, the colorectal cancer patients in the training set (COAD and READ) were partitioned into two definitive subgroups. The optimal clustering number was determined as K = 2, substantiated by Figures 3A, B and Supplementary Figures S1A–C. The patient distribution was 506 in cluster 1 and 140 in cluster 2, with the DE-LRGs’ expression patterns displayed in Figure 3C. Notably, survival analyses, as reflected by the K-M plot, did not identify significant differences between these clusters (Supplementary Figures S2A–C).

### Distinct tumor microenvironments and mutational landscapes characterize the molecular subtypes

**FIGURE 4:**
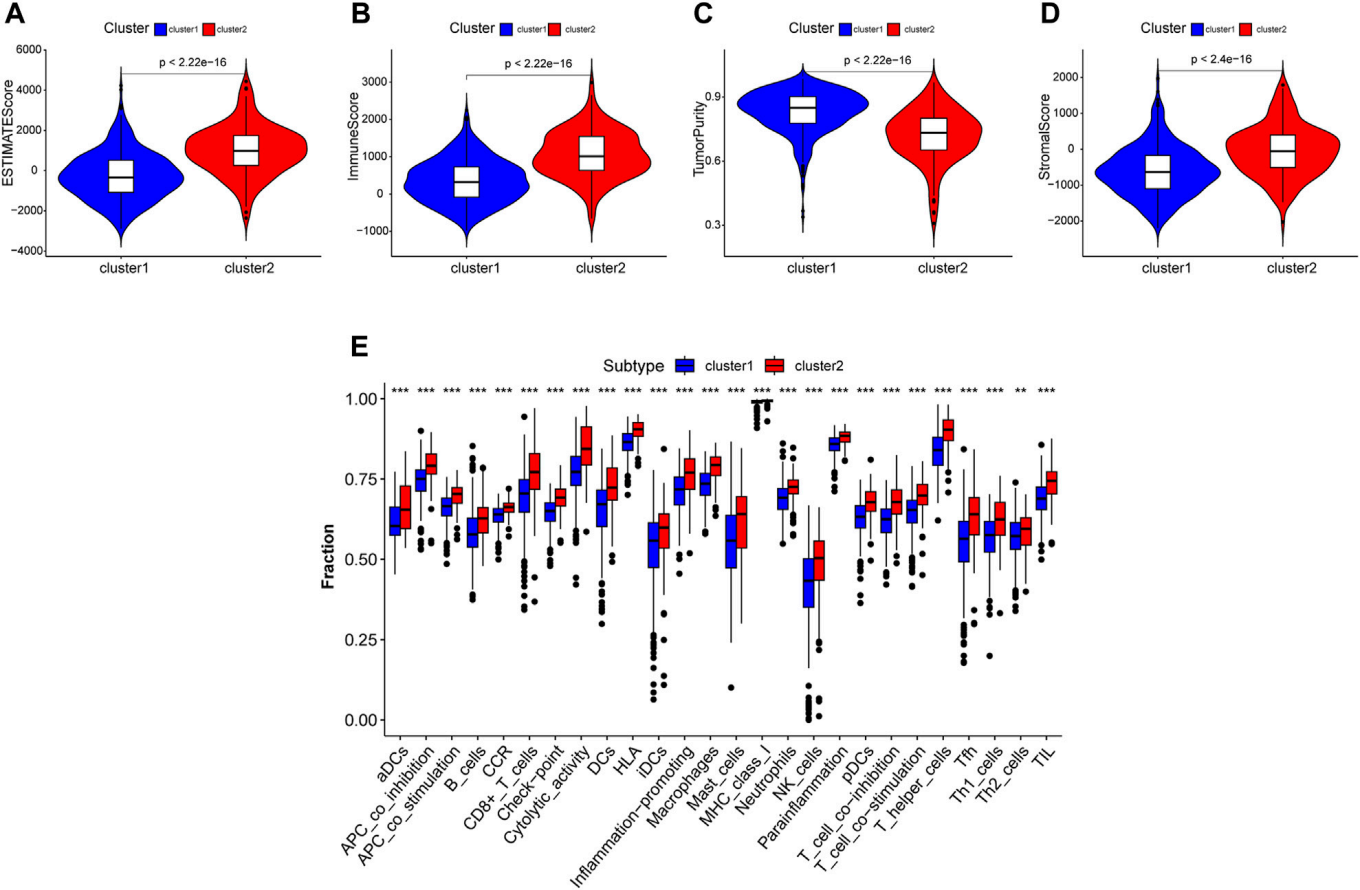
Tumor microenviroment analysis in different clustered subtypes. **(A–D)** Estimate score, immuoscore, stromal score and tumor purity were calculated. Except tumor purity, Estimate score, immunoscore and stromal score of cluster 2 were all higher than cluster 1. **(E)** Immune cell infiltration levels in two subtypes analysed by ssGSEA algorithm respectively.

A comprehensive immune analysis was employed to delineate the differential immune and mutational profiles of the two molecular subtypes. The ESTIMATE algorithm unveiled pronounced disparities, with cluster 2 manifesting significantly augmented ESTIMATE (*p* < 0.0001), Immunocore (*p* < 0.0001), and stromal scores (*p* < 0.0001) juxtaposed with a diminished tumor purity (*p* < 0.0001) compared to cluster 1 (Figures 4A–D).

An expansive immune landscape evaluation via the ssGSEA algorithm further underscored the pronounced divergence between clusters, with all 26 immune cell types exhibiting marked enrichment in cluster 2 (Figure 4E).

### Functional pathway analysis reveals distinctive characteristics between clusters

**FIGURE 5:**
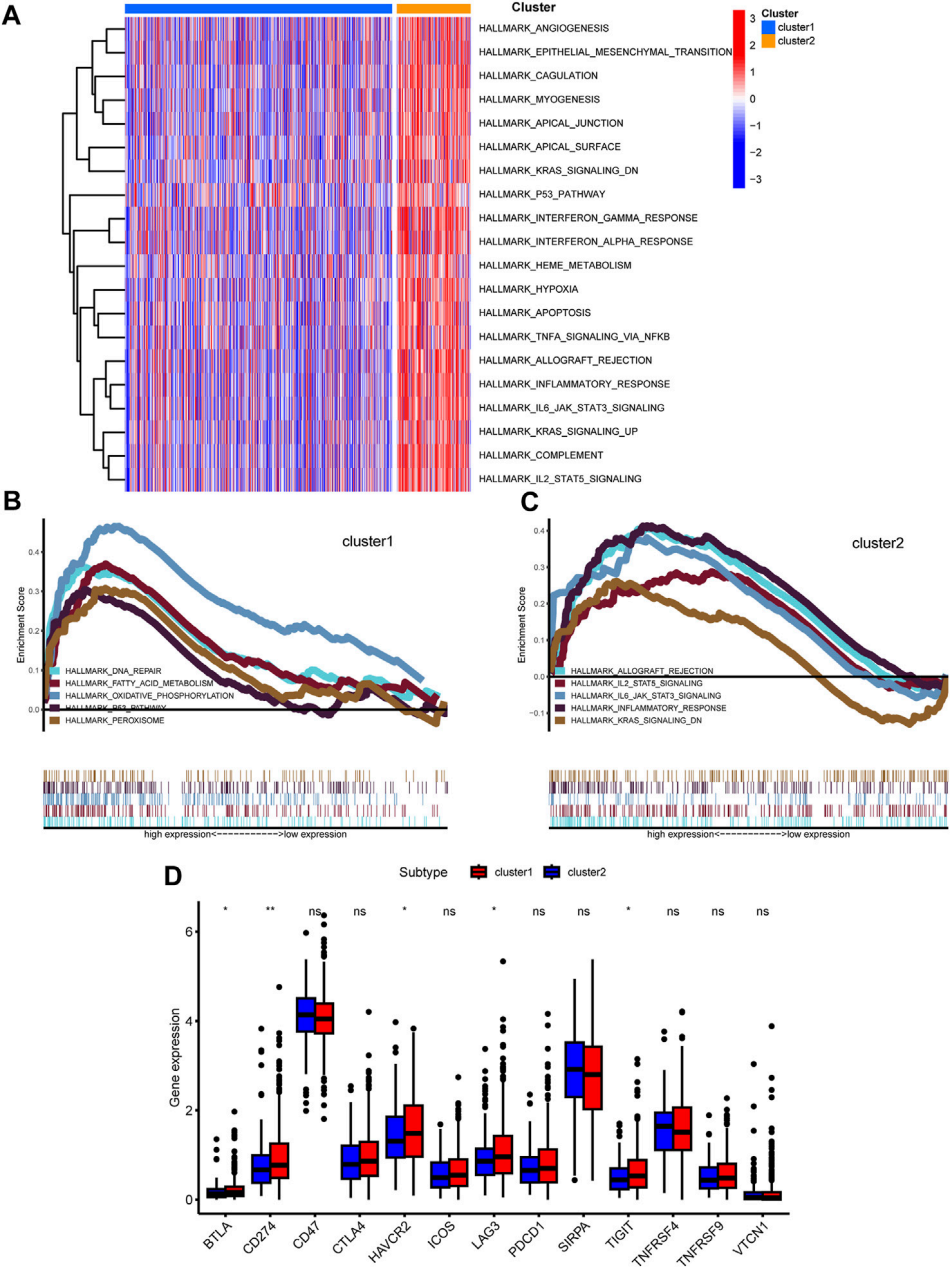
Functional analysis among two clusters. **(A)** Heatmap shown enriched differential pathways by GSVA. **(B,C)** Different functions in two clusters were enriched by GSEA. **(D)** Expression of immunue checkpoint-related genes was shown.

To discern the enriched pathways and pivotal functions that segregate colorectal patients across clusters, both GSVA and GSEA were harnessed. The GSVA highlighted a panoply of divergent pathways (Figure 5A). Specifically, pathways integral to angiogenesis, epithelial-mesenchymal transition, coagulation, and several signaling cascades, including KRAS, p53, interferon, TNF, and inflammatory response, were markedly accentuated in cluster 2 relative to cluster 1. Furthermore, GSEA delineated distinctive pathways: while cluster 1 was enriched in DNA repair, fatty acid metabolism, and oxidative phosphorylation, cluster 2 manifested heightened IL-2/STAT5, IL-6/JAK/STAT3 signaling, and KRAS signaling activities (Figures 5B, C). Intriguingly, the differential expression of immune checkpoint-associated genes was evident between clusters, with a set of genes including BTLA, CD200R1, and CD276, among others, showing amplified expression in cluster 2 (Figure 5D).

### Risk model development and validation

**FIGURE 6:**
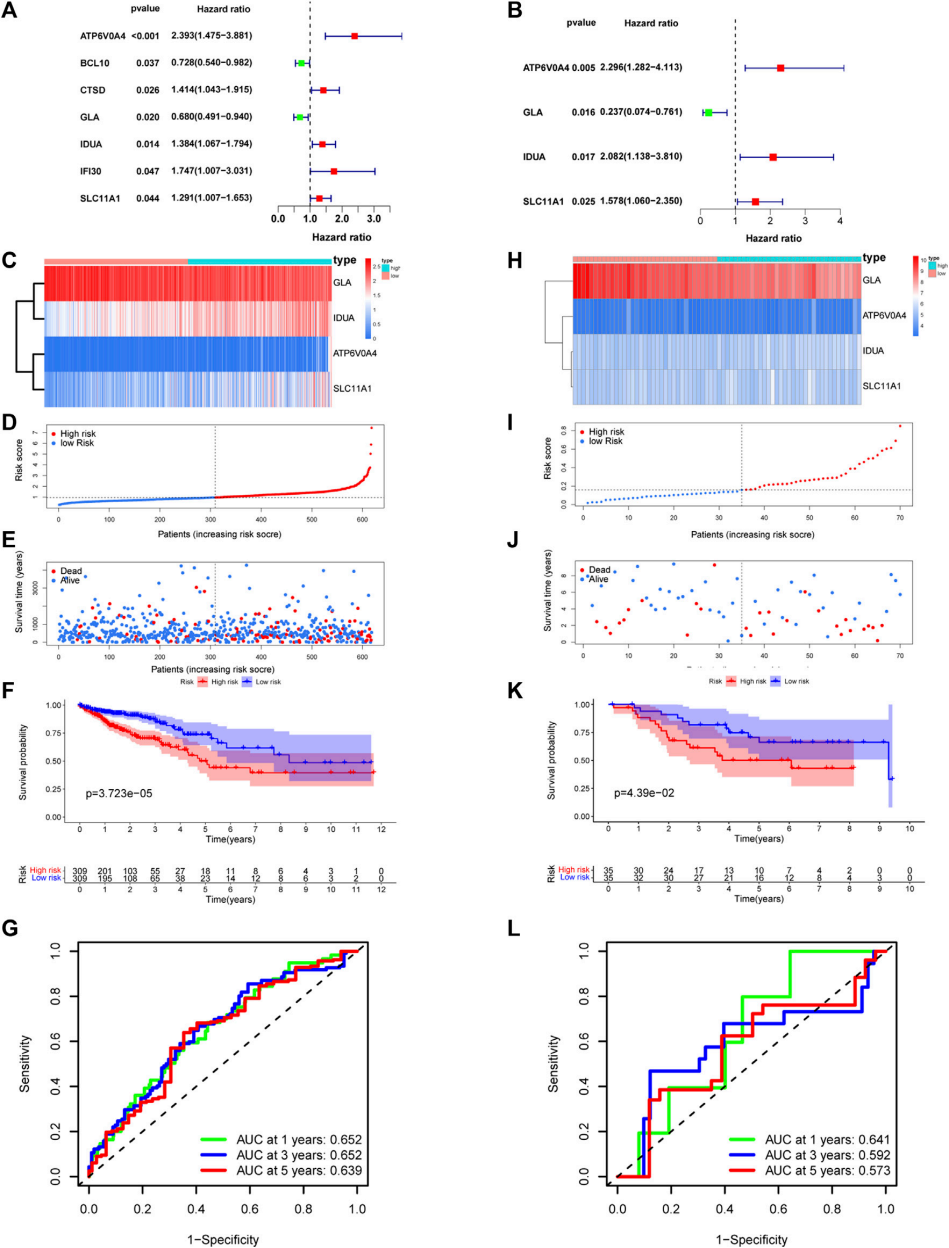
Establishment and validation of risk model. **(A,B)** Univariate-multivariate Cox analysis were conducted to screened prognosis-related genes. Four risk related DE-LRGs included ATP6V0A4, GLA, IDUA and SLC11A1 were screened. **(C)** Expression level of risk related DE-LRGs among different risk groups. **(D,E)** Distribution of risk score and survival time of CRC patients in high and low risk groups. **(F)** Survival curve of the CRC patients in different risk group. **(G)** Time-dependent ROC curve of the risk model. **(H–L)** Similar result was obtained in verification group.

Utilizing univariate Cox analysis, prognosis-associated genes from DE-LRGs were identified. The subsequent multivariate Cox regression discerned ATP6V0A4, GLA, IDUA, and SLC11A1 as pivotal for the risk model, as evidenced in the forest plot (Figures 6A, B). Risk scores were then deduced for each patient across training and validation cohorts using the formula: risk score= (0.831×expression value of ATP6V0A4) + (−1.44×expression value of GLA) + (0.733×expression value of IDUA)+ (0.456×expression value of SLC11A1). This resultant model adeptly partitioned patients into high- and low-risk factions (Figures 6C–E). A heatmap further elucidated the differential expression of central genes across these risk groups (Figures 6C–E). Alarmingly, those within the high-risk category confronted a more dire survival outcome than their low-risk peers (Figure 6F). The ROC curve attested to the model’s commendable predictive accuracy (Figure 6G), a finding echoed in the validation dataset (Figures 6H–L).

### Risk gene-immune cell correlation and experimental validation

**FIGURE 7:**
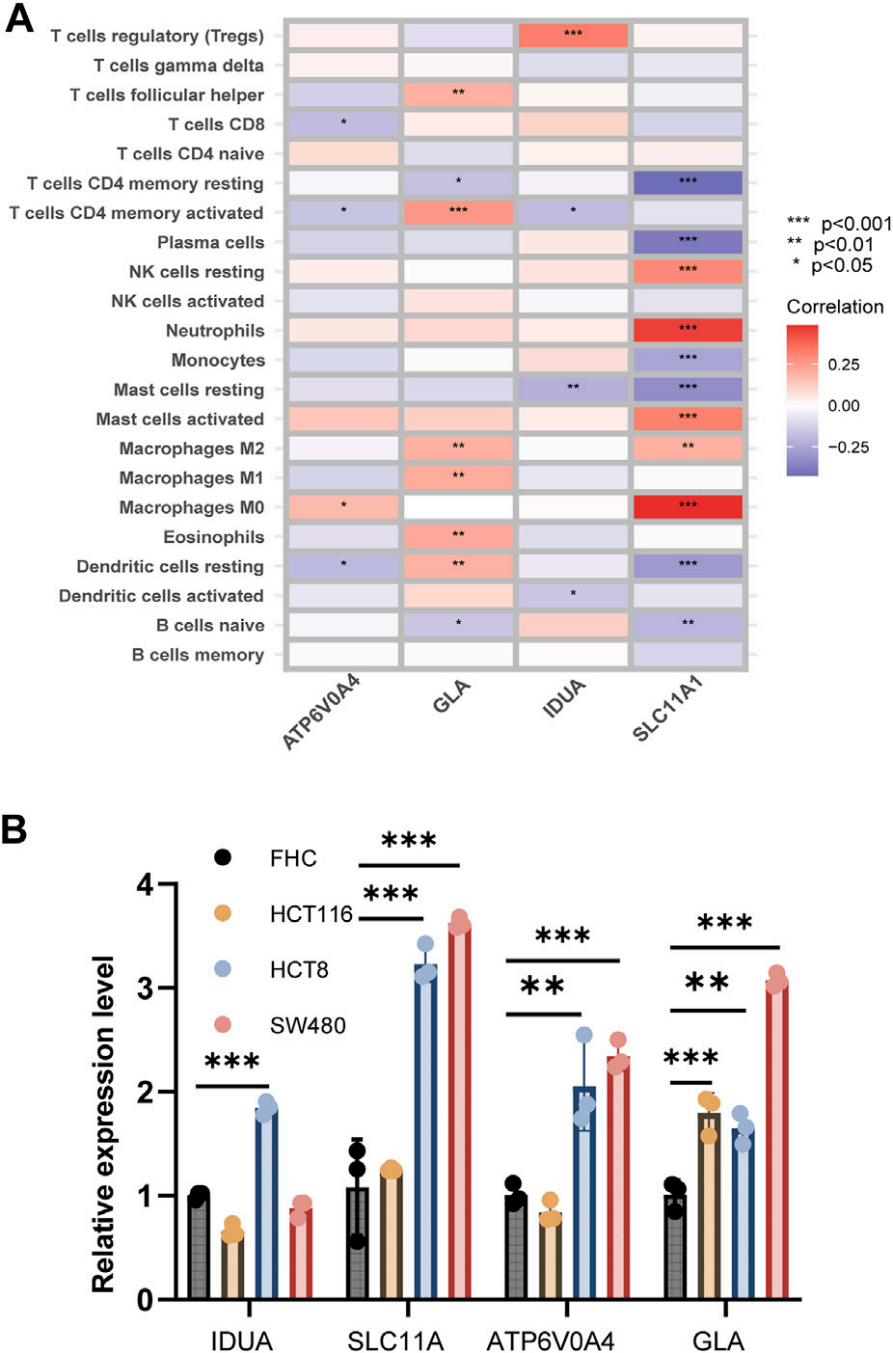
Correlation analysis between risk genes and immune cells. **(A)** The heatmap shown correlation between risk genes and immune cells. **(B)** Expression level of four risk related DE-LRGs in cell line.

Figure 7A elucidates the intricate associations between risk genes and immune cell populations. Notably, ATP6V0A4 expression manifested positive ties with M0 macrophages and negative ties with CD8^+^ T cells, CD4^+^ memory-activated T cells, and dormant dendritic cells. GLA’s expression correlated positively with subsets like M1 and M2 macrophages, and CD4^+^ memory-activated T cells, but negatively with CD4^+^ memory resting T cells and naive B cells. IDUA’s expression exhibited affinities with Treg cells, but displayed discord with resting mast cells, CD4^+^ memory-activated T cells, and active dendritic cells. SLC11A1’s expression showed positive relations with various cells, including resting NK cells and M0 macrophages, but negative with cells like naive B cells and resting dendritic cells.

Employing qRT-PCR, we gauged the expression of the four risk-associated DE-LRGs across normal and tumor-derived colon cell lines (Figure 7B). Remarkably, IDUA expression surged in HCT8, while both SLC11A1 and ATP6V0A4 were significantly upregulated in HCT8 and SW480 relative to FHC. GLA’s expression was notably augmented across all tumor cell lines.

### Independence of the constructed risk model and built a novel nomogram model

**FIGURE 8:**
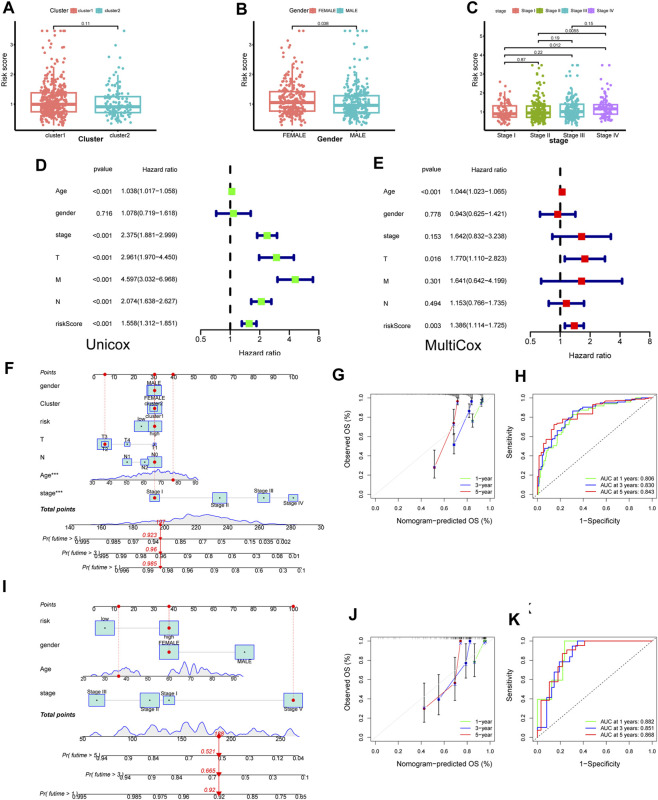
Construction of novel risk model and nomogram. **(A–C)** The association between risk score and clinical features (cluster, gender and stage). **(D,E)** Forest plot to show the result of Univariate and Multivariate Cox regression. **(F)** Nomogram integrating risk score and clinical features based on TCGA training dataset. **(G)** Calibration of the nomogram at 1,3 and 5 years in the training cohort. **(J,H)** Predictive efficiency of the nomogram was shown by ROC curve. **(I–K)** Nomogram built based on validation corhort (GSE39084) and its calibration curve and ROC curve.

Probing deeper, we assessed the interplay between the risk score and clinical attributes, substantiating the independence of our formulated risk model via subgroup and regression analyses (Figures 8A–C and Supplementary Figures S2F–I). Intriguingly, female patients and those with advanced or metastatic colorectal cancer exhibited heightened risk scores. Univariate-multivariate Cox regression analyses further solidified our risk model as a standalone prognostic indicator for colorectal cancer (Figures 8D, E).

Capitalizing on these insights, we sculpted a cutting-edge nomogram for prognosticating CRC outcomes (Figure 8F). For instance, consider a 78-year-old female patient with stage I CRC; her cumulative score of 197 translates to a 1-year survival likelihood of ∼98.5%, and a 5-year survival rate nearing 92.3%. The ROC curve accentuated the model’s precision, with the calibration curve affirming a tight alignment between forecasted and actual overall survival rates (Figures 8G, H). Parallel findings were replicated utilizing a GEO validation dataset, underscoring the model’s robustness (Figures 8I–K).

### Risk score: Interplay with oncogenic activity, tumor mutation burden, and immunological insights

**FIGURE 9:**
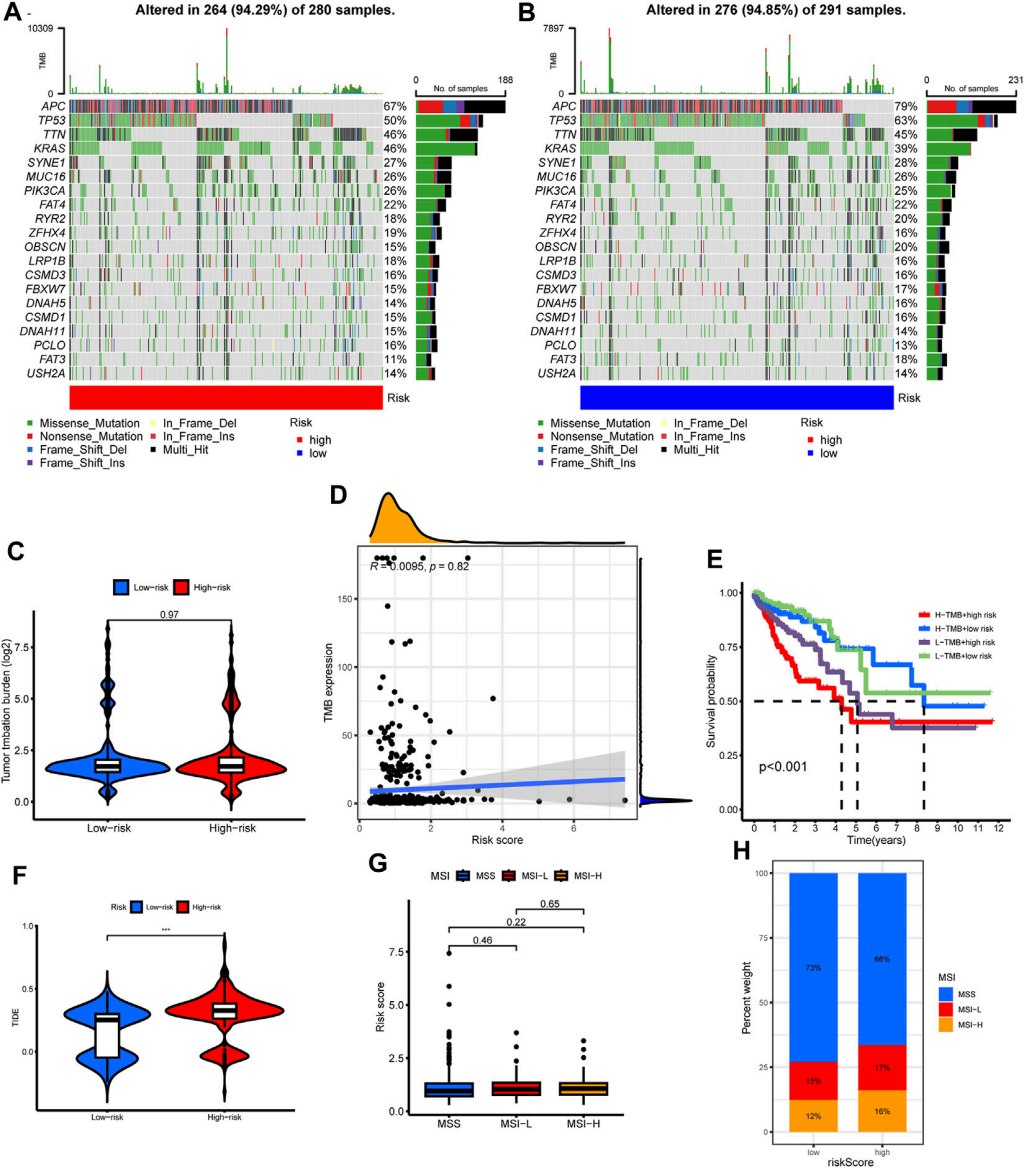
The correlations of tumor mutation with risk score, cluster subtypes and survival. **(A,B)** The oncoprint map displayed the top 20 most prevalent genes in high-risk and low-risk groups. **(C)** TMB level among different risk group. **(D)** Correlation analysis among TMB level and risk score. **(E)** Survival analysis combined with TMB and risk score. **(F)** Differences in TIDE score level among different risk group. **(G)** Difference in risk scores among different microsatellite status. **(H)** Composition of microsatellite status in low and high-risk groups.

We explored the risk score’s genomic dimensions by assessing gene mutations. The oncoprint highlighted the 20 most prevalent mutated genes in both risk categories, with missense mutations predominantly featured. While APC mutations were ubiquitous, they showed differential prevalence: 79% in low-risk versus 67% in the high-risk group (Figures 9A,B).

Regarding tumor mutation burden (TMB), no discernible difference spanned the risk groups (Figure 9C). The risk score and TMB also lacked significant correlation (Figure 9D). Although the high TMB group did not showcase a survival advantage (Supplementary Figure S2E), an intriguing pattern emerged when juxtaposing TMB with risk scores: a combination of high TMB and low-risk scores portended favorable prognoses (Figure 9E).

Leveraging the TIDE algorithm, we discerned elevated TIDE scores in the high-risk cohort (Figure 9F). However, microsatellite status comparisons between risk groups remained non-significant (Figures 9G, H).

### Targeted therapeutic efficacy & immunotherapeutic responsiveness assessment

**FIGURE 10:**
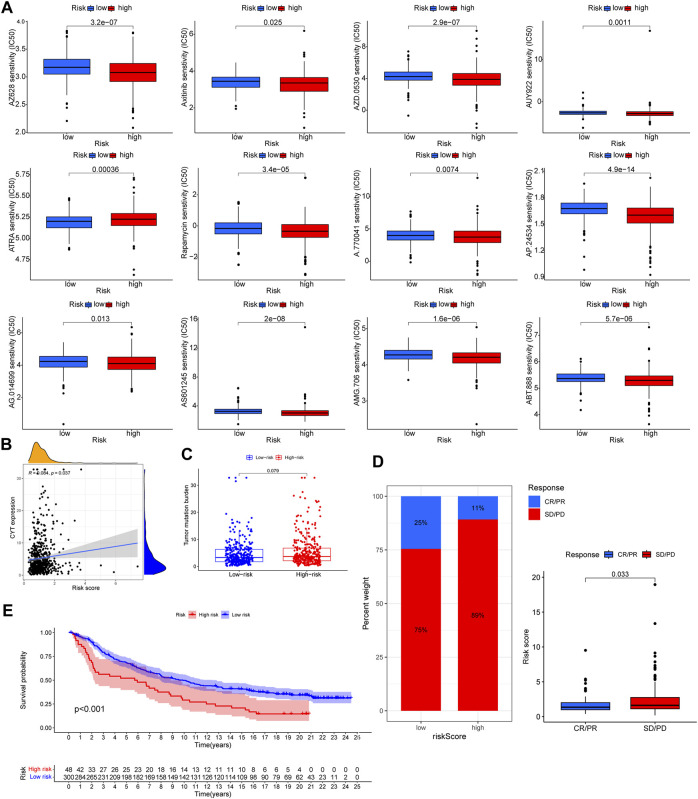
The correlation of targeted therapeutic and immunotherapeutic effectiveness with risk score. **(A)** Significant relationship between risk score and targeted therapy sensitivity used pRRophetic algorithm. **(B)** Significantly positive correlation among CYT and risk score. **(C)** TMB level among different risk group. No significant difference wae observed. **(D)** Patients with SD/PD had higher risk score. **(E)** Patients treated with immunotherapy in high risk group had poorer prognosis. Supplementary Table S1 List of lysosomes-related genes.

The pRRophetic algorithm was harnessed to unearth the nexus between risk scores and targeted therapeutic susceptibilities. Remarkably, the low-risk category displayed enhanced sensitivity to a spectrum of inhibitors, barring ATRA (Figure 10A).

Distinct correlations emerged between the CYT score and risk score, but TMB differences across risk groups were inconsequential (Figures 10B, C). Employing the IMvigor210 cohort to gauge immunotherapy responsiveness, we observed that a lower risk score corresponded with enhanced prognosis and immunotherapeutic sensitivity (Figures 10D, E).

## Discussion

Colorectal cancer persists as a formidable global health challenge. Even with advancements like targeted and immunotherapies, achieving a marked improvement in patient prognosis remains an unmet goal. This underscores an imperative for adept risk stratification and tailored therapeutic interventions. Intriguingly, lysosome-associated genes have been spotlighted as pivotal players in tumorigenesis.

Within this study’s framework, we discerned two distinct molecular clusters and subsequently forged a novel risk model, anchored in the differential expression of lysosome-associated genes. Notably, patients within the high-risk spectrum were predominantly aligned with the second cluster. These individuals were characterized by elevated ESTIMATE and immune scores, enhanced immune cell infiltration, and a pronounced TMB. However, they exhibited diminished tumor purity. A pattern emerged wherein patients with less favorable prognoses frequently exhibited elevated risk metrics juxtaposed with a diminished TMB.

Delving deeper, our functional analysis illuminated a nexus between the augmented anti-tumor responses and oncogenic pathways, especially evident with increased immune cell infiltration within the tumor milieu. Further, our therapeutic efficacy evaluation of both targeted and immunotherapies highlighted an augmented treatment responsiveness in low-risk patients. Collectively, these insights have the potential to reshape colorectal cancer therapeutic paradigms and guide clinicians towards more informed decision-making.

Leveraging consensus clustering, we adeptly delineated patients into distinct molecular cohorts, informed by the expression profile of DE-LRGs. While the overall survival between these cohorts remained statistically congruent, discernible variations became evident in the tumor-immune interplay (TIME), risk metrics, and TMB. Our investigations further shed light on the role of DE-LRGs in shaping colorectal cancer dynamics.

TIME plays a pivotal role in determining tumor evolution and shaping patient prognosis. Pioneering algorithms, namely, ESTIMATE, have emerged as tools to deduce tumor purity and the interplay of immune and stromal cells within tumors based on gene expression (Mao et al., 2018; Gong et al., 2020; Deng et al., 2021). The ESTIMATE algorithm, in particular, quantifies immune components within tumor specimens, offering a mirror to TIME dynamics. A salient feature is tumor purity, delineated as the proportion of malignant entities within tumor tissue, and its association with prognosis is undeniable. Prevailing research corroborates our findings wherein diminished tumor purity often aligns with adverse prognoses (Pages et al., 2018; Mlecnik et al., 2020), while heightened immune metrics portend favorable outcomes in colorectal malignancies. Moreover, the multifaceted role of diverse immune cell infiltrates in tumorigenesis has been accentuated (Grivennikov et al., 2010; Greten and Grivennikov, 2019; Mao et al., 2021). Within our investigative paradigm, tools like ESTIMATE were harnessed to profile the TIME across clusters, revealing pronounced immune metrics but diminished tumor purity in the second cluster. Further, the deployment of ssGSEA unveiled nuances of immune status, emphasizing the predominance of specific immune-related entities. Our observations aligned well with extant literature, underscoring the heightened immune landscape evident in the second cluster.

In a subsequent phase of our investigation, we devised an innovative risk model rooted in the differential expression of lysosome-related genes (DE-LRGs), with its validity affirmed within an external cohort. Leveraging both uni- and multi-Cox regression modalities, we pinpointed risk-pertinent DE-LRGs integrally associated with tumor ontogeny and progression. Notably, ATP6V0A4, a constituent of the v-ATPases ensemble, has been documented to manifest pronouncedly in breast cancer and gliomas (Hinton et al., 2009; Gleize et al., 2012; Savci-Heijink et al., 2019). Glaucocalyxin A, denoted as GLA, has been recognized for its tumor-suppressive capacities across a gamut of malignancies (Zhu et al., 2018; Chen et al., 2021). While IDUA is traditionally linked with Hurler syndrome, contemporary research illuminates its upregulated presence in renal and breast carcinomas (Osborn et al., 2008; Xing et al., 2021). SLA11A1, colloquially termed Nramp1, is a member of the solute carrier lineage with roles in metal homeostasis (Montalbetti et al., 2013; Zhu et al., 2022). Its accentuated expression in CRC not only portends an unfavorable prognosis but also earmarks its potential as a predictor for immunotherapeutic responsiveness (Ma et al., 2022).

Our survival assessments underscored the formidable predictive capacity of this risk model, consistently so across both foundational and validation cohorts. Progressing further, we sculpted a nomogram that harmoniously integrated risk indices with clinical parameters, demonstrating robust prognostic and predictive finesse. A meticulous examination of patient demographics across the clusters manifested a preponderance of low-risk entities within cluster 2. Despite this, overarching survival trajectories remained statistically indistinct between the cohorts. Synthesizing these insights, it emerges cogently that an enriched immune milieu, characterized by augmented immune cell recruitment, underpins the diminished risk profile observed within cluster 2.

To discern the intrinsic biological distinctions between the two clusters, we employed GSVA and GSEA for comprehensive functional analyses. Using GSVA, we quantified the signaling pathway activities within individual samples derived from gene expression profiles, identifying a pronounced augmentation of these activities in cluster 2. Through GSEA, an integrative tool for analyzing gene expression data, we precisely delineated gene set expression patterns across the clusters. Notably, our GSEA findings highlighted a predilection for immune response-associated pathways in cluster 2, while pathways tethered to oncogenes, oxidative distress, and DNA repair were accentuated in cluster 1. This molecular landscape offers clarity on the elevated risk disposition observed predominantly among patients in cluster 1.

In the final phase of our analysis, we appraised the efficacy of targeted and immunotherapeutic interventions using the pRRophetic algorithm, TIDE, and IMvigor210. The pRRophetic algorithm facilitated the estimation of targeted therapy efficacy, unveiling enhanced responsiveness in low-risk patients across all targeted agents. Concurrently, we assessed the efficacy of immunotherapy leveraging both TIDE and IMvigor210. Previous literature underscores that elevated TIDE scores correlate with unfavorable prognosis and heightened immunotherapy resistance (Jiang et al., 2018; Qiu et al., 2021; Shi et al., 2021). Consistent with this, our data revealed that patients within the high-risk cohort manifested superior TIDE scores, suggesting augmented resistance to immunotherapy among these CRC patients. Further substantiation was sought from survival and efficacy data from the IMvigor210 repository. Intriguingly, CRC patients undergoing PD-1 inhibitor treatment exhibited a distinct dichotomy: those with lower risk scores demonstrated superior survival trajectories, and individuals displaying robust responsiveness to immunotherapy consistently harbored significantly diminished risk scores compared to their non-responsive counterparts.

While our investigation sheds light on the intriguing role of lysosome-related genes in CRC, it is imperative to acknowledge certain constraints. Notably, despite patients in cluster 2 manifesting traits typically associated with a more favorable prognosis—higher immune scores and reduced risk scores—there was not a significant disparity in overall survival when juxtaposed against cluster 1. This observation could stem from the relatively diminutive sample size of cluster 2. Furthermore, our conclusions, derived predominantly from bioinformatics analyses, necessitate corroboration through rigorous experimental endeavors. It is also pertinent to note that our dataset, sourced from open-access repositories, beckons validation in more expansive patient cohorts to definitively ascertain the clinical implications of lysosome-related genes in CRC.

## Conclusion

In CRC, we delineated DE-LRGs into distinct clusters, with ATP6V0A4, GLA, IDUA, and SLC11A1 pivotal to risk assessment. Cluster 2 displayed enhanced anti-tumor immunity and favorable prognosis. Significantly, low-risk patients showed enhanced treatment susceptibility, underscoring the clinical promise of these DE-LRGs in CRC management.

## Data Availability

The original contributions presented in the study are included in the article/Supplementary Material, further inquiries can be directed to the corresponding authors.

## References

[B1] Andre T. Meyerhardt J. Iveson T. Sobrero A. Yoshino T. Souglakos I. (2020). Effect of duration of adjuvant chemotherapy for patients with stage III colon cancer (IDEA collaboration): final results from a prospective, pooled analysis of six randomised, phase 3 trials. Lancet Oncol. 21, 1620–1629. 10.1016/S1470-2045(20)30527-1 33271092 PMC7786835

[B2] Ballabio A. Bonifacino J. S. (2020). Lysosomes as dynamic regulators of cell and organismal homeostasis. Nat. Rev. Mol. Cell. Biol. 21, 101–118. 10.1038/s41580-019-0185-4 31768005

[B3] Biller L. H. Schrag D. (2021). Diagnosis and treatment of metastatic colorectal cancer: a review. JAMA 325, 669–685. 10.1001/jama.2021.0106 33591350

[B4] Chen Y. H. Xu N. Z. Hong C. Li W. Q. Zhang Y. Q. Yu X. Y. (2022). Myo1b promotes tumor progression and angiogenesis by inhibiting autophagic degradation of HIF-1α in colorectal cancer. Cell. Death Dis. 13, 939. 10.1038/s41419-022-05397-1 36347835 PMC9643372

[B5] Chen J. Zhang W. Pan C. Fan J. Zhong X. Tang S. (2021). Glaucocalyxin A induces cell cycle arrest and apoptosis via inhibiting NF-κB/p65 signaling pathway in melanoma cells. Life Sci. 271, 119185. 10.1016/j.lfs.2021.119185 33577846

[B6] Dekker E. Tanis P. J. Vleugels J. L. A. Kasi P. M. Wallace M. B. (2019). Colorectal cancer. Lancet 394, 1467–1480. 10.1016/S0140-6736(19)32319-0 31631858

[B7] Deng Y. Song Z. Huang L. Guo Z. Tong B. Sun M. (2021). Tumor purity as a prognosis and immunotherapy relevant feature in cervical cancer. Aging (Albany NY) 13, 24768–24785. 10.18632/aging.203714 34844217 PMC8660621

[B8] Diaz L. A. Shiu K. K. Kim T. W. Jensen B. V. Jensen L. H. Punt C. (2022). Pembrolizumab versus chemotherapy for microsatellite instability-high or mismatch repair-deficient metastatic colorectal cancer (KEYNOTE-177): final analysis of a randomised, open-label, phase 3 study. Lancet Oncol. 23, 659–670. 10.1016/S1470-2045(22)00197-8 35427471 PMC9533375

[B9] Fu Y. Gu Q. Luo L. Xu J. Luo Y. Xia F. (2020). New anti-cancer strategy to suppress colorectal cancer growth through inhibition of ATG4B and lysosome function. Cancers (Basel) 12, 1523. 10.3390/cancers12061523 32532053 PMC7352571

[B10] Ganesh K. Stadler Z. K. Cercek A. Mendelsohn R. B. Shia J. Segal N. H. (2019). Immunotherapy in colorectal cancer: rationale, challenges and potential. Nat. Rev. Gastroenterol. Hepatol. 16, 361–375. 10.1038/s41575-019-0126-x 30886395 PMC7295073

[B11] Geeleher P. Cox N. Huang R. S. (2014). pRRophetic: an R package for prediction of clinical chemotherapeutic response from tumor gene expression levels. PLoS One 9, e107468. 10.1371/journal.pone.0107468 25229481 PMC4167990

[B12] Gleize V. Boisselier B. Marie Y. Poea-Guyon S. Sanson M. Morel N. (2012). The renal v-ATPase a4 subunit is expressed in specific subtypes of human gliomas. Glia 60, 1004–1012. 10.1002/glia.22332 22460948

[B13] Gong Z. Zhang J. Guo W. (2020). Tumor purity as a prognosis and immunotherapy relevant feature in gastric cancer. Cancer Med. 9, 9052–9063. 10.1002/cam4.3505 33030278 PMC7724479

[B14] Gou Q. Dong C. Xu H. Khan B. Jin J. Liu Q. (2020). PD-L1 degradation pathway and immunotherapy for cancer. Cell. Death Dis. 11, 955. 10.1038/s41419-020-03140-2 33159034 PMC7648632

[B15] Greten F. R. Grivennikov S. I. (2019). Inflammation and cancer: triggers, mechanisms, and consequences. Immunity 51, 27–41. 10.1016/j.immuni.2019.06.025 31315034 PMC6831096

[B16] Grivennikov S. I. Greten F. R. Karin M. (2010). Immunity, inflammation, and cancer. Cell. 140, 883–899. 10.1016/j.cell.2010.01.025 20303878 PMC2866629

[B17] Hinton A. Sennoune S. R. Bond S. Fang M. Reuveni M. Sahagian G. G. (2009). Function of a subunit isoforms of the V-ATPase in pH homeostasis and *in vitro* invasion of MDA-MB231 human breast cancer cells. J. Biol. Chem. 284, 16400–16408. 10.1074/jbc.M901201200 19366680 PMC2713521

[B18] Jiang P. Gu S. Pan D. Fu J. Sahu A. Hu X. (2018). Signatures of T cell dysfunction and exclusion predict cancer immunotherapy response. Nat. Med. 24, 1550–1558. 10.1038/s41591-018-0136-1 30127393 PMC6487502

[B19] Mao X. Xu J. Wang W. Liang C. Hua J. Liu J. (2021). Crosstalk between cancer-associated fibroblasts and immune cells in the tumor microenvironment: new findings and future perspectives. Mol. Cancer 20, 131. 10.1186/s12943-021-01428-1 34635121 PMC8504100

[B20] Mao Y. Feng Q. Zheng P. Yang L. Liu T. Xu Y. (2018). Low tumor purity is associated with poor prognosis, heavy mutation burden, and intense immune phenotype in colon cancer. Cancer Manag. Res. 10, 3569–3577. 10.2147/CMAR.S171855 30271205 PMC6149864

[B21] Mariathasan S. Turley S. J. Nickles D. Castiglioni A. Yuen K. Wang Y. (2018). TGFβ attenuates tumour response to PD-L1 blockade by contributing to exclusion of T cells. Nature 554, 544–548. 10.1038/nature25501 29443960 PMC6028240

[B22] Ma Y. Zhan L. Yang J. Zhang J. (2022). SLC11A1 associated with tumor microenvironment is a potential biomarker of prognosis and immunotherapy efficacy for colorectal cancer. Front. Pharmacol. 13, 984555. 10.3389/fphar.2022.984555 36438826 PMC9681808

[B23] Mayakonda A. Lin D. C. Assenov Y. Plass C. Koeffler H. P. (2018). Maftools: efficient and comprehensive analysis of somatic variants in cancer. Genome Res. 28, 1747–1756. 10.1101/gr.239244.118 30341162 PMC6211645

[B24] Mlecnik B. Bifulco C. Bindea G. Marliot F. Lugli A. Lee J. J. (2020). Multicenter international society for immunotherapy of cancer study of the consensus immunoscore for the prediction of survival and response to chemotherapy in stage III colon cancer. J. Clin. Oncol. 38, 3638–3651. 10.1200/JCO.19.03205 32897827 PMC7605397

[B25] Montalbetti N. Simonin A. Kovacs G. Hediger M. A. (2013). Mammalian iron transporters: families SLC11 and SLC40. Mol. Asp. Med. 34, 270–287. 10.1016/j.mam.2013.01.002 23506870

[B26] Osborn M. J. Mcelmurry R. T. Peacock B. Tolar J. Blazar B. R. (2008). Targeting of the CNS in MPS-IH using a nonviral transferrin-alpha-L-iduronidase fusion gene product. Mol. Ther. 16, 1459–1466. 10.1038/mt.2008.119 PMC2574880 18523448

[B27] Pages F. Mlecnik B. Marliot F. Bindea G. Ou F. S. Bifulco C. (2018). International validation of the consensus Immunoscore for the classification of colon cancer: a prognostic and accuracy study. Lancet 391, 2128–2139. 10.1016/S0140-6736(18)30789-X 29754777

[B28] Perera R. M. Zoncu R. (2016). The lysosome as a regulatory hub. Annu. Rev. Cell. Dev. Biol. 32, 223–253. 10.1146/annurev-cellbio-111315-125125 27501449 PMC9345128

[B29] Piao S. Amaravadi R. K. (2016). Targeting the lysosome in cancer. Ann. N. Y. Acad. Sci. 1371, 45–54. 10.1111/nyas.12953 26599426 PMC4879098

[B30] Qiu C. Shi W. Wu H. Zou S. Li J. Wang D. (2021). Identification of molecular subtypes and a prognostic signature based on inflammation-related genes in colon adenocarcinoma. Front. Immunol. 12, 769685. 10.3389/fimmu.2021.769685 35003085 PMC8733947

[B31] Rizzollo F. More S. Vangheluwe P. Agostinis P. (2021). The lysosome as a master regulator of iron metabolism. Trends Biochem. Sci. 46, 960–975. 10.1016/j.tibs.2021.07.003 34384657

[B32] Rooney M. S. Shukla S. A. Wu C. J. Getz G. Hacohen N. (2015). Molecular and genetic properties of tumors associated with local immune cytolytic activity. Cell. 160, 48–61. 10.1016/j.cell.2014.12.033 25594174 PMC4856474

[B33] Savci-Heijink C. D. Halfwerk H. Koster J. Horlings H. M. van de Vijver M. J. (2019). A specific gene expression signature for visceral organ metastasis in breast cancer. BMC Cancer 19, 333. 10.1186/s12885-019-5554-z 30961553 PMC6454625

[B34] Shi J. Bao M. Wang W. Wu X. Li Y. Zhao C. (2021). Integrated profiling identifies PLOD3 as a potential prognostic and immunotherapy relevant biomarker in colorectal cancer. Front. Immunol. 12, 722807. 10.3389/fimmu.2021.722807 34646265 PMC8503557

[B35] Torre L. A. Bray F. Siegel R. L. Ferlay J. Lortet-Tieulent J. Jemal A. (2015). Global cancer statistics, 2012. CA Cancer J. Clin. 65, 87–108. 10.3322/caac.21262 25651787

[B36] Wang Y. du J. Wu X. Abdelrehem A. Ren Y. Liu C. (2021). Crosstalk between autophagy and microbiota in cancer progression. Mol. Cancer 20, 163. 10.1186/s12943-021-01461-0 34895252 PMC8665582

[B37] Wilkerson M. D. Hayes D. N. (2010). ConsensusClusterPlus: a class discovery tool with confidence assessments and item tracking. Bioinformatics 26, 1572–1573. 10.1093/bioinformatics/btq170 20427518 PMC2881355

[B38] Xing Q. Zeng T. Liu S. Cheng H. Ma L. Wang Y. (2021). A novel 10 glycolysis-related genes signature could predict overall survival for clear cell renal cell carcinoma. BMC Cancer 21, 381. 10.1186/s12885-021-08111-0 33836688 PMC8034085

[B39] Yang S. T. Fan J. B. Liu T. T. Ning S. Xu J. H. Zhou Y. J. (2022). Development of strigolactones as novel autophagy/mitophagy inhibitors against colorectal cancer cells by blocking the autophagosome-lysosome fusion. J. Med. Chem. 65, 9706–9717. 10.1021/acs.jmedchem.2c00275 35852796

[B40] Yoshihara K. Shahmoradgoli M. Martinez E. Vegesna R. Kim H. Torres-Garcia W. (2013). Inferring tumour purity and stromal and immune cell admixture from expression data. Nat. Commun. 4, 2612. 10.1038/ncomms3612 24113773 PMC3826632

[B41] Zhao C. Qiu S. He J. Peng Y. Xu H. Feng Z. (2020). Prodigiosin impairs autophagosome-lysosome fusion that sensitizes colorectal cancer cells to 5-fluorouracil-induced cell death. Cancer Lett. 481, 15–23. 10.1016/j.canlet.2020.03.010 32184145

[B42] Zhu J. Sun Y. Lu Y. Jiang X. Ma B. Yu L. (2018). Glaucocalyxin A exerts anticancer effect on osteosarcoma by inhibiting GLI1 nuclear translocation via regulating PI3K/Akt pathway. Cell. Death Dis. 9, 708. 10.1038/s41419-018-0684-9 29899333 PMC5999605

[B43] Zhu Q. Meng Y. Li S. Xin J. du M. Wang M. (2022). Association of genetic variants in autophagy-lysosome pathway genes with susceptibility and survival to prostate cancer. Gene 808, 145953. 10.1016/j.gene.2021.145953 34500048

