# Supplementary material for: Computational profiling and prognostic modeling based on lysosome-related genes in colorectal cancer
Source: Front Genet. 2023 Nov 23;14:1203035. doi: 10.3389/fgene.2023.1203035 (PMC10701274; doi:10.3389/fgene.2023.1203035)
Supplement: Supplementary file 1 [file Table1.DOCX]

Supplement table1

| Lysosomes  related  genes | Gene List |
| --- | --- |
|  | ABCA2 ACR ADRB2 ANKRD27 AP3M1ARSB AZU1 BCL10 KNL1CD63 CLN5 CORO1A CTNS CTSC CTSF CYLC1 DNASE2 GALC GLA HLA-DOB HLA-DRA HPS1 HPS4 HYAL2 IFI30 CXCR2 KCNE1 KCNE2 LAMP2 LAMP3 LIPA LRP2 MPO MYO7A NAGLU NEU4 NPC2 PPT1 RAB14 RAB7A RAB9A RAMP2 RAMP3 SFTPD SLC17A5 SPACA3 SRGN STS TIAL1 TOM1 TOM1L1 TPP1 TRIM23 TSPAN8 TYR USE1 USP4 USP5 USP6 VAMP4 VPS45 AP1M2 AP3S2 TCIRG1 AP4B1AP3M2 AP4S1 AP1S1 AP3S1CLN3 CLTA CLTB CLTC AP1S3 HGSNAT CTSB CTSD CTSE CTSH CTSK CTSL CTSV CTSO CTSS CTSW CTSZ AP1B1 AP1G1AGA ARSG GGA2GGA3 AP4E1ABCB9 ATP6V0A2 PLA2G15 ATP6VOD2 FUCA1 GAA MFSD8 GALNS GGA1GBA GLB1GM2A GNS SUMF1 GUSB HEXA HEXB HYAL1 IDS IDUA IGF2R LAMP1 M6PR ARSA MAN2B1 MANBA ASAH1 NAGA NEU1 NPC1 SLC11A2 ATP6VOA4 NAGPA ATP6V1H ATP6VOC ACP2 ATP6VOB ATP6VOA1 ATP6AP1 ACP5 CTSA LAPTM4B LGMN PSAP MCOLN1 DNASE2B SORT1 SGSH SLC11A1 SMPD1 PSAPL1 LAPTM5 GNPTAB AP3B2 CLTCL1 GNPTG AP3B1 CD164 AP1S2 AP1M1 AP3D1 ATP6VOD1 AP4M1PPT2 NAPSA SCARB2 ENTPD4 CD68 LAPTM4A |


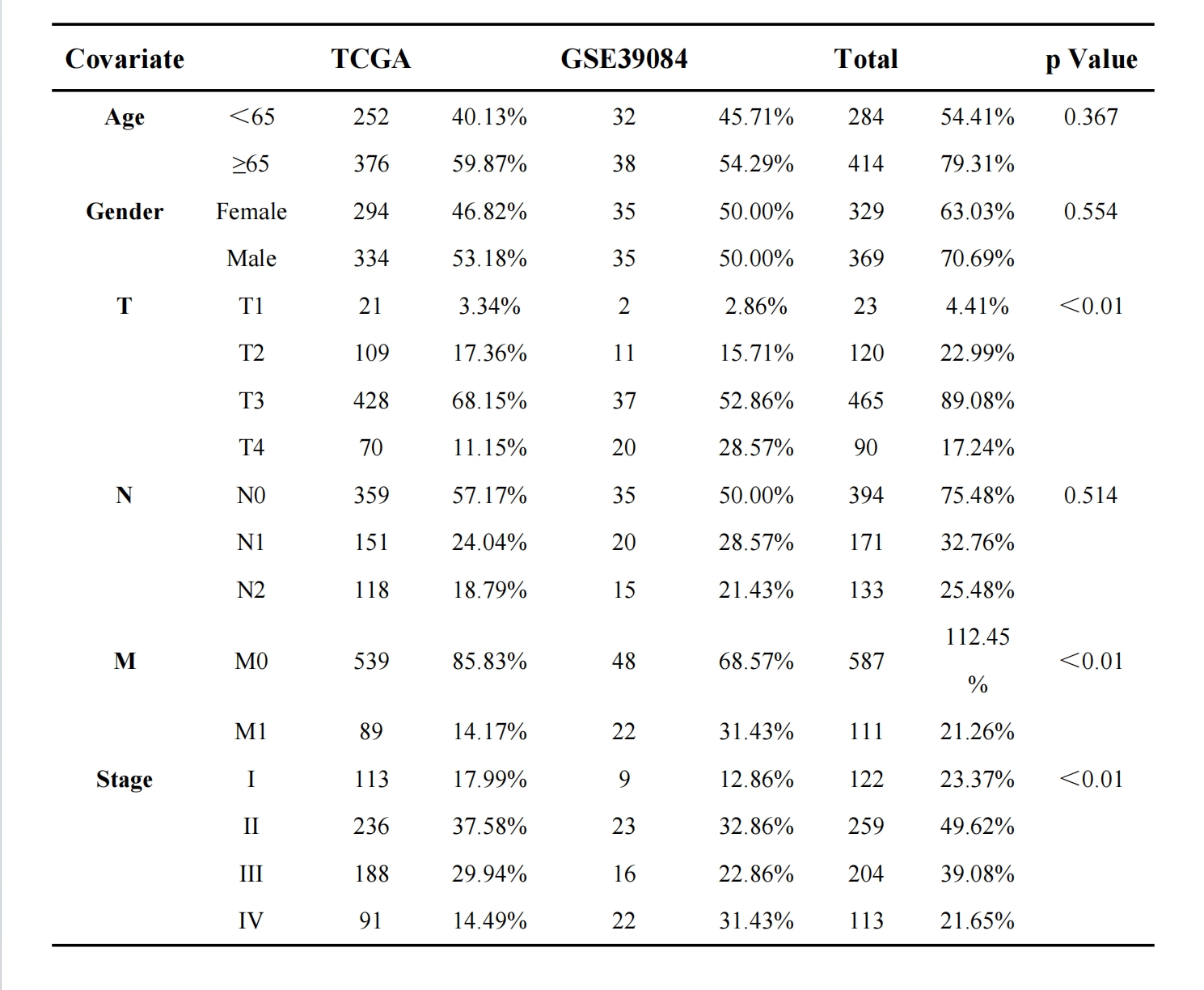
Supplement table2
