# Supplementary figures and images for: Computational profiling and prognostic modeling based on lysosome-related genes in colorectal cancer
Source: Front Genet. 2023 Nov 23;14:1203035. doi: 10.3389/fgene.2023.1203035 (PMC10701274; doi:10.3389/fgene.2023.1203035)

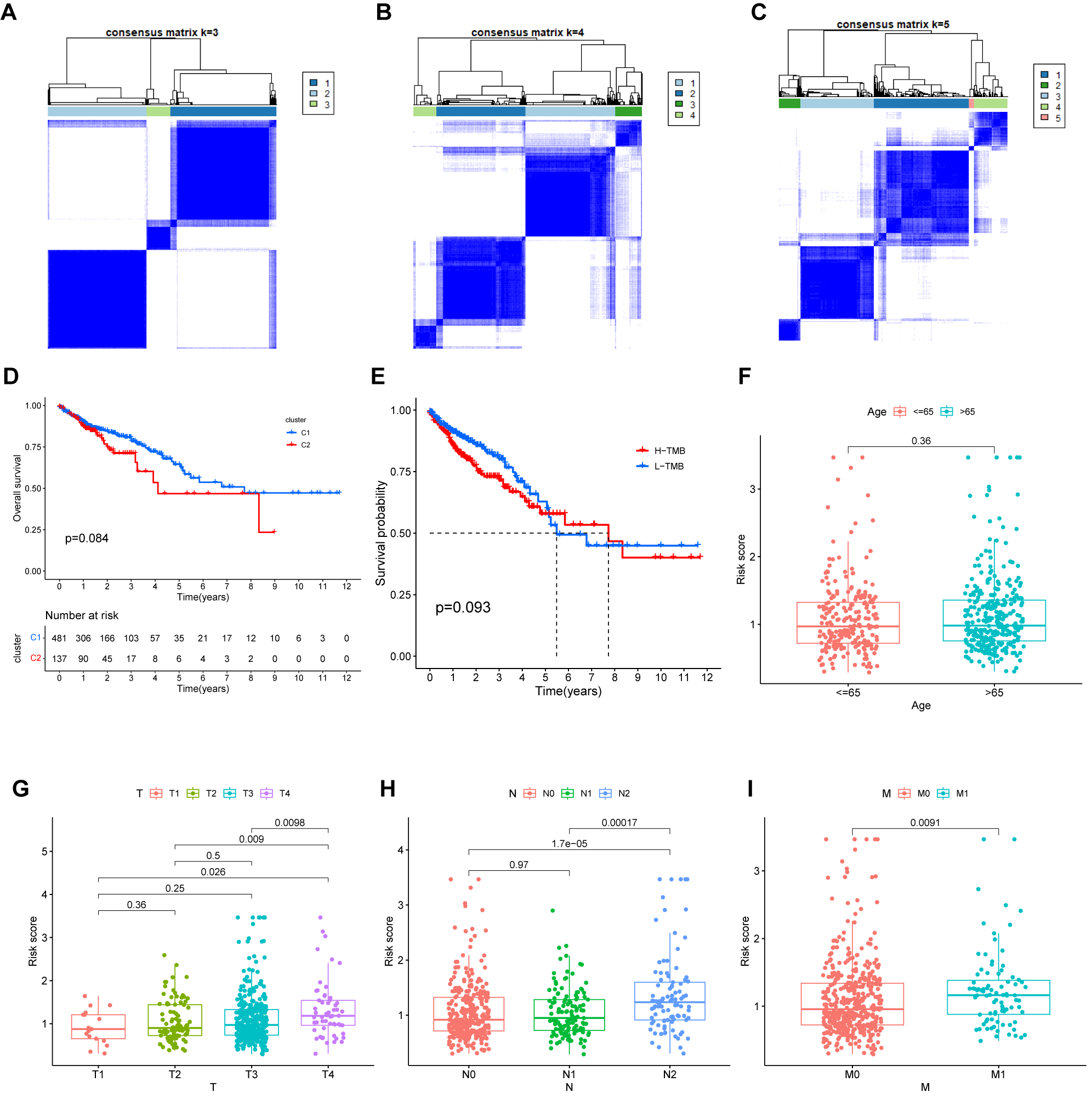

Supplement: Supplementary file 2 [file Image2.TIF]

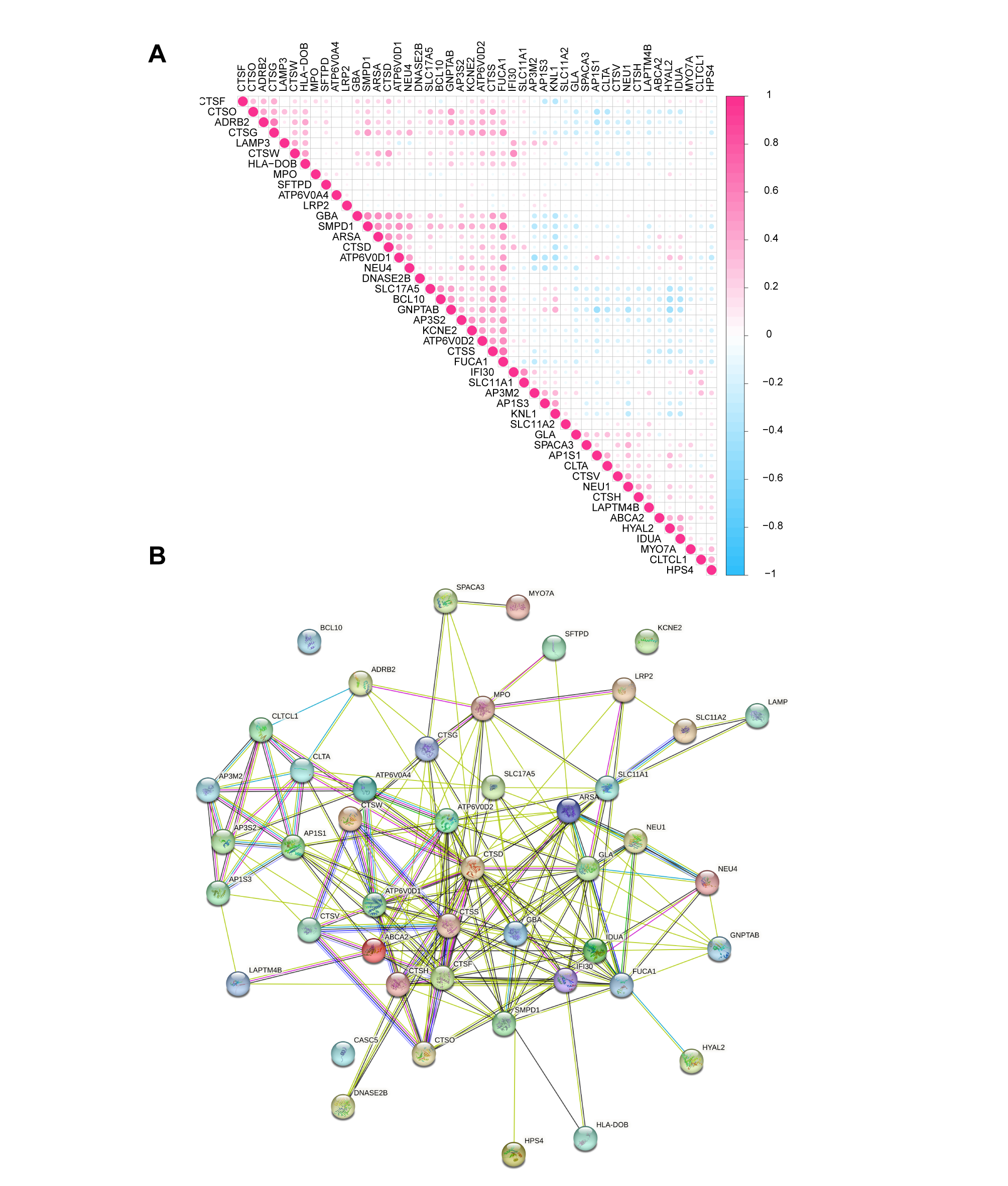

Supplement: Supplementary file 3 [file Image1.TIF]
